# Development, Survival and Reproduction of *Nezara viridula* (Hemiptera: Pentatomidae) in Sesame Cultivars and Implications for the Management

**DOI:** 10.3390/plants13081060

**Published:** 2024-04-09

**Authors:** Adrielly Karoliny de Lima, José Janduí Soares, Marcus Alvarenga Soares, José Cola Zanuncio, Carla de Lima Bicho, Carlos Alberto Domingues da Silva

**Affiliations:** 1 Departamento de Ciências Biológicas, Universidade Estadual da Paraíba, Avenida das Baraúnas, 351, Campina Grande 58429-500, PB, Brazil; adriellyklima@gmail.com (A.K.d.L.); clbicho@servidor.uepb.edu.br (C.d.L.B.); 2 Embrapa Algodão, Rua Oswaldo Cruz, 1143, Campina Grande 58428-095, PB, Brazil; jose-jandui.soares@embrapa.br; 3 Departamento de Agronomia, Universidade Federal dos Vales do Jequitinhonha e Mucuri, Diamantina 39100-000, MG, Brazil; marcus.alvarenga@ufvjm.edu.br; 4 Departamento de Entomologia/BIOAGRO, Universidade Federal de Viçosa, Viçosa 36570-900, MG, Brazil; zanuncio@ufv.br

**Keywords:** feeding effect, host plant resistance, *Sesamum indicum*, stinkbug

## Abstract

Sesame, an oilseed plant with multiple applications, is susceptible to infestations by the stink bug *Nezara viridula* (Linnaeus, 1758) (Hemiptera: Pentatomidae). This pest suctions the seeds of this plant and injects toxins into them. Possible sources of resistance on sesame cultivars are important to manage this bug. The objective of this study was to evaluate the biological aspects of *N. viridula* fed on three sesame cultivars aiming to select possible resistance sources for integrated pest management (IPM) programs of this stinkbug. The experimental design used randomized blocks with three treatments and four replications, each with newly emerged *N. viridula* nymphs fed with sesame capsules of the cultivars BRS Anahí (T1), BRS Morena (T2) and BRS Seda (T3). Two to three green sesame capsules were supplied every two days per group of ten *N. viridula* nymphs as one replication until the beginning of the adult stage. Adults of this stinkbug were fed in the same manner as its nymphs but with mature sesame capsules until the end of the observations. Survival during each of the five instars and of the nymph stage of *N. viridula* with green sesame capsules was similar between cultivars, but the duration of the nymph stage was shorter with green capsules of the BRS Morena than with those of the BRS Anahí. The oviposition period, number of egg masses and eggs per female, and the percentage of nymphs hatched were higher with mature capsules of the sesame cultivar BRS Anahí and lower with the others. Nymphs did not hatch from eggs deposited by females fed mature seed capsules of the sesame cultivar BRS Morena, which may indicate a source of resistance against this stinkbug in this cultivar. The worldwide importance of *N. viridula* to sesame cultivation makes these results useful for breeding programs of this plant aiming to develop genotypes resistant to this bug. In addition, the BRS Morena is a cultivar already commercially available and can be recommended in places where there is a history of incidence of *N. viridula*, aiming to manage the populations of this pest.

## 1. Introduction

Most sesame cultivars (*Sesamum indicum* L., Pedaliaceae), one of the oldest oilseed plants in the world, originated from sub-Saharan Africa [1]. This plant is cultivated mainly in the tropics and subtropics, with Argentina, China, Colombia, Egypt, Greece, El Salvador, India, Mexico, Myanmar, Nicaragua, Pakistan, Sudan, Turkey, the USA, and Venezuela being the main producers [2]. The states of Ceará, Mato Grosso, Mato Grosso do Sul and Pará [3], with an estimated area of 148 thousand hectares and production of approximately 99,000 tons in the 2021/2022 crop, concentrate the sesame production in Brazil [4].

The nutritional quality of the seeds, for humans, and its medicinal purposes makes sesame one of the most important cultivated plants in the world [5]. The area cultivated with this plant in the semi-arid region of Northeast Brazil is small, although its high rusticity, resistance to drought and great economic use indicate suitability for cultivation in this region [3]. Sesame seeds are rich in oil (50–62%), with high and low levels of unsaturated and saturated essential fatty acids, respectively, in addition to proteins (18–25%), carbohydrates (13.4–25.0%) and digestible fibers (9.8%) [6], making this plant attractive and vulnerable to insect pests [7,8].

Around 38 arthropod species damage sesame plants [7,8] with losses, due to insects reaching 25–30% of their yield, especially during the flowering stage [8,9]. The leaf-rollerworm *Antigastra catalaunalis* (Duponchel, 1833) (Lepidoptera: Pyralidae); the aphids *Aphis* sp. and *Myzus persicae* (Sulzer, 1776) (Hemiptera: Aphididae); the whiteflies *Bemisia argentifolii* (Bellows and Perring, 1997) and *Bemisia tabaci* (Gennadius, 1889) (Hemiptera: Aleyrodidae); the green leafhopper *Empoasca* sp. (Hemiptera: Cicadellidae), the leafcutters *Atta* spp. (Hymenoptera: Formicidae) [10]; and the green stinkbug, *Nezara viridula* (Linnaeus, 1758) (Hemiptera: Pentatomidae) are the main insect pests of sesame in Brazil [11].

The stinkbug *N. viridula*, widely distributed across the American continent, sucks seeds from the beans of soybean and sesame and from their fruit structures [12]. The nymphs and adults of *N*. *viridula* uses piercing–sucking mouthparts to feed on and injure seeds and seed capsules, causing stains and reducing the number and quality of seeds, oil and germination rate, in addition to increasing protein content and altering plant physiology [13].

Resistant cultivars, an ideal method to manage pest populations, can reduce the number of *N. viridula* individuals below economic levels without pollution in the agroecosystem and additional problems to the farmer [14,15,16]. Furthermore, the enhanced production of crop plants resistant to insects is a key component of the required response to two major challenges of the 21st century—increased food production and decreased CO_2_ emissions driving climate change. Specifically, global food production must increase dramatically by 2050 to feed a projected 30% population increase, while attempts must continue to be made to reduce CO_2_ emissions related to insecticide production and use [17].

Genetic breeding is necessary to evaluate the resistance of *S. indicum* to the green stinkbug *N. viridula*. The commercial sesame cultivars BRS Seda, BRS Anahí and BRS Morena, available through the Embrapa Algodão genetic improvement program, are the most recently developed ones [18]. Morphophysiological characteristics, such as color, hairiness and oil content in their seeds, may be resistance sources of these cultivars to *N. viridula* [13,19,20].

The introgression of exogenous genes for insect resistance is difficult, time consuming, and expensive in commercial sesame plants. The selection of proteins with pesticide action and derived from *Bacillus thuringiensis* (Bt), which kill sap-sucking hemipterans, is scarce [21,22] with most efforts made to manage insect chewing pests [21,23]. Therefore, classical genetic improvement, aiming to obtain plants resistant to sap-sucking insects, especially *N. viridula*, is important because it can be a reduced or even substitutive alternative to insecticides applied to sesame crops. Furthermore, it presents other advantages such as ease of access and reduction in cultivation costs without interfering with the agroecosystem, making the crop more profitable for the producer.

The objective was to evaluate biological aspects of *N. viridula* to select possible resistance sources in the sesame cultivars BRS Anahí, BRS Morena and BRS Seda for use in the integrated pest management (IPM) of this stinkbug.

## 2. Results

**Figure 1:**
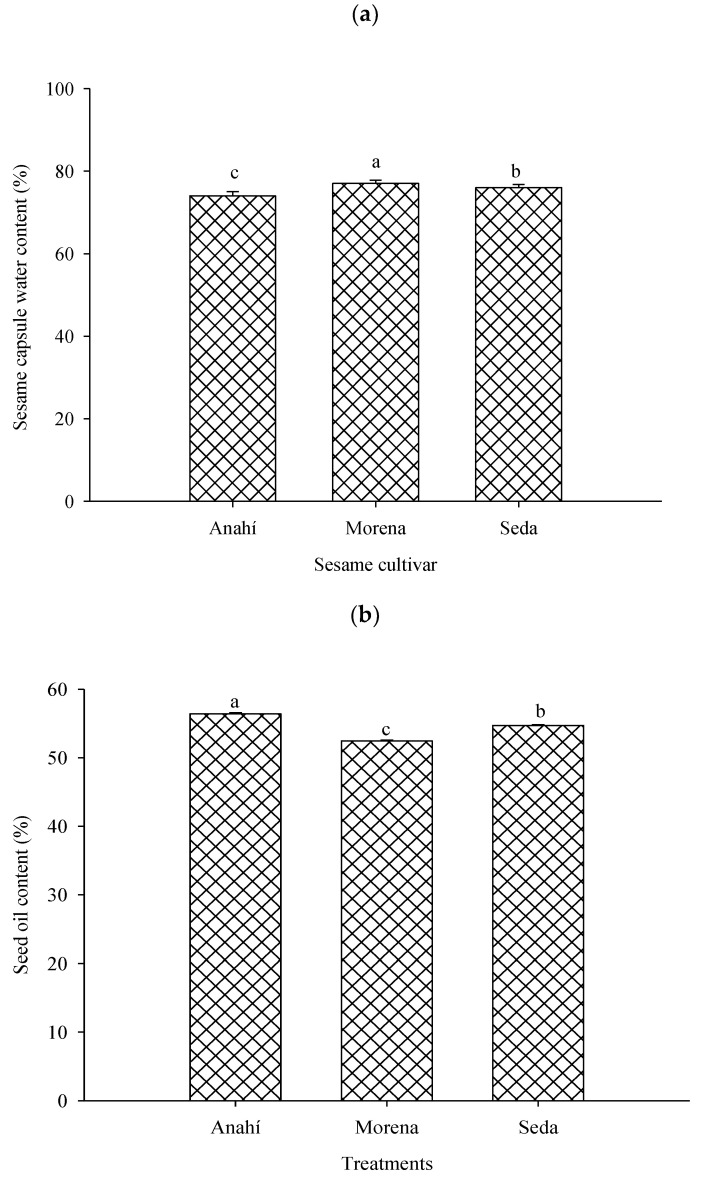
Water (**a**) and oil (**b**) content, respectively, of immature capsules and mature seeds of sesame (*Sesamum indicum* L.) cultivars BRS Anahi, BRS Morena and BRS Seda. Bars followed by the same lowercase letter per treatment do not differ according to the Tukey test at 5% probability. Campina Grande, Paraíba state, Brazil, 2023.

The water content of the green seed capsules of the cultivar BRS Morena was higher than that of the other ones and its seed oil content the lowest. The water and seed oil content of the BRS Anahi capsule were the lowest and highest, respectively (Figure 1).

The survival of *N. viridula* varied between its instars, but was similar between sesame cultivars and without interactions between these parameters (Table 1). The duration of the nymph stage of *N. viridula* varied between cultivars and instars, but without interaction between these factors.

The survival during each instar and of the total nymph stage did not differ between sesame cultivars. However, the survival per instar and of the total nymph stage was similar but with the highest value in the first, second and third (Table 2).

The period of the nymph stage and of each instar of *N. viridula* was longer with the immature capsules of the BRS Anahí sesame cultivar than with those of the BRS Morena. The duration of the first and fifth instars of *N. viridula* was longest with the three sesame cultivars (Table 2).

**Figure 2:**
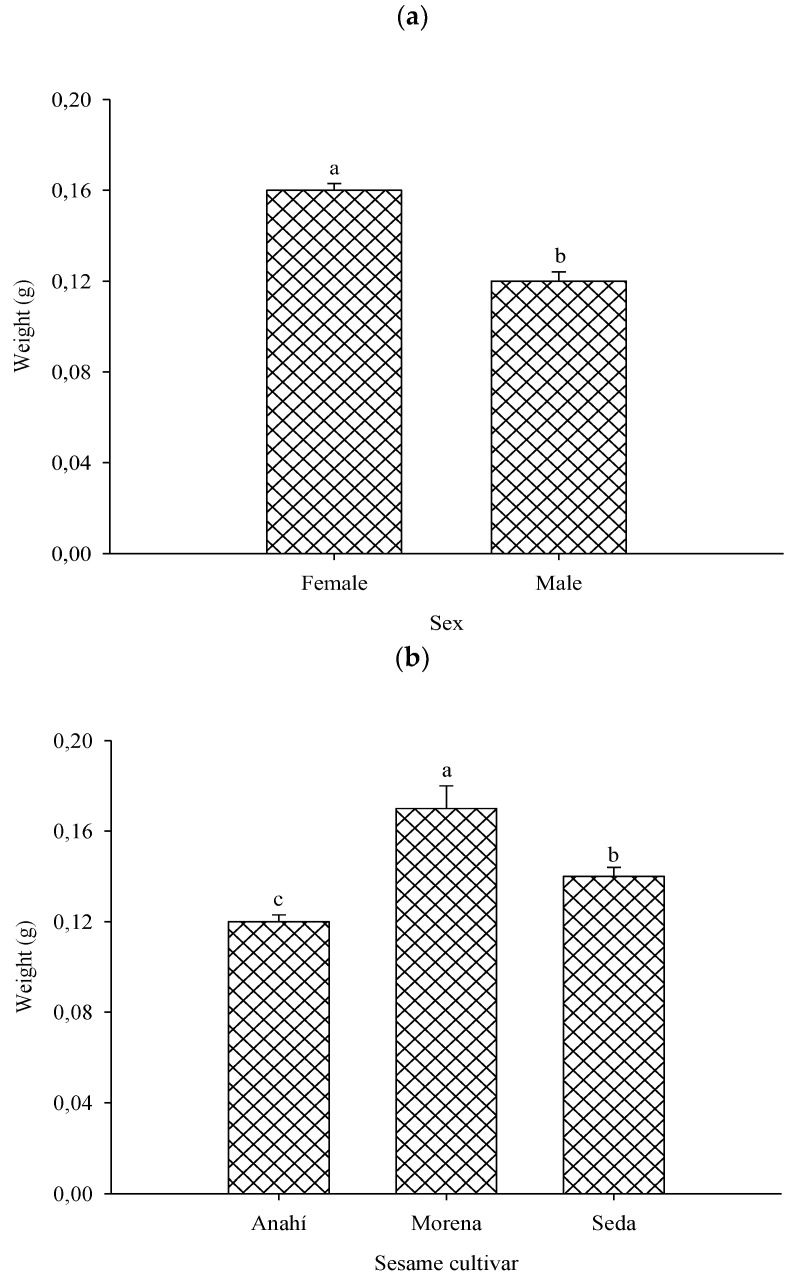
Weight of *Nezara viridula* (Hemiptera: Pentatomidae) adults fed with immature sesame capsules (fruits) of the cultivars BRS Anahi, BRS Morena and BRS Seda per sex (a) and cultivar (**b**). Campina Grande, Paraíba state, Brazil, 2023.

*Nezara viridula* females were heavier than males in all treatments (Figure 2A) and individuals of both sexes of this bug were also heavier with the immature capsules of the sesame cultivars of BRS Morena, followed by those of BRS Seda and lighter with those of BRS Anahí (Figure 2B).

The pre-oviposition and oviposition periods of *N. viridula* were longer with mature sesame capsules of the BRS Morena and shorter with those of the BRS Anahí, and the pre-oviposition and oviposition periods were shorter with the BRS Anahí and the BRS Morena and BRS Seda, respectively. Post-oviposition was similar between the sesame cultivars (Table 3).

The number of egg masses and eggs per female of *N. viridula* was higher when fed with mature capsules of the sesame cultivar BRS Anahí (Table 3) and lower with those of the BRS Morena and BRS Seda. The number of eggs per egg mass of *N. viridula* females was higher when fed with capsules of the BRS Morena and BRS Anahí sesame cultivars than with those of BRS Seda. The number of nymphs hatched per female of *N. viridula* was higher with mature capsules of the BRS Anahí sesame cultivar (Table 3) than with those of BRS Seda and BRS Morena, without hatching with the latter one.

The longevity of *N. viridula* adults, fed with mature sesame capsules, was similar between sexes (Table 3) and longer with BRS Anahí and BRS Morena than with BRS Seda.

## 3. Discussion

Genetic and phenotypic variations may explain the higher water and lower oil content in the seeds and green capsules, respectively, of the sesame BRS Morena and BRS Anahí [24]. This is important because these substances may affect nutrient intake by *N. viridula* nymphs and adults [20,25]. Furthermore, the affinity of seeds with high oil content for water is low and, therefore, as the oil content increases, that of water decreases [26].

Variations in the survival of *N. viridula* per instar and in the duration of its nymph stage between sesame cultivars are probably due to morphophysiological and behavioral differences between the instars of this stinkbug and the water content of the green sesame capsules [25,27]. The bundle of nymph stylets, in the initial instars of *N. viridula*, is shorter than in more advanced ones, making it necessary for the first ones to group, facilitating access to the mesocarp of the sesame capsules, which may explain the higher survival in these instars [27,28]. In the first instar, the potential penetration depth of the stylet is in the range of 120.6 to 291.3 μm, complicating the access to the endocarp in capsules with thick and fully developed mesocarp (parenchyma), but not in those parts of the capsule at the ends with a narrower mesocarp [29,30]. This explains the preference of *N. viridula* nymphs to aggregate at the end of the first instar in locations to increase the access to the endocarp of the sesame capsules [27]. These results are similar to those for nymphs of this stink bug fed with green bean pods [29]. However, this behavior tends to disappear as the nymphs develop into their final instars [28]. Furthermore, first-instar *N. viridula* nymphs feed on the endocarp of immature sesame capsules to ingest sap and maintain body hydration [27,29,30]. Research using rifampicin-resistant marked bacteria showed definitively that first-instar *N. viridula* probed into and ingested liquid from cut segments of green beans [29,30].

The similar survival during the nymph stage and in the first, second, third, fourth and fifth instars of *N. viridula* fed with immature capsules of the three sesame cultivars may be related to the physical and chemical attributes (nutrients and non-nutrients) from these reproductive plant structures [27,31]. These results were similar to those of *N. viridula* fed immature sesame capsules of an unknown variety [11]. Differences in the nymph gregarious behavior, solitary from the third instar, may explain the greater and lower survival of first and third instar nymphs, respectively, of *N. viridula* [29].

The longer duration of the nymph stage and per instar of *N. viridula* fed with immature sesame capsules of the BRS Anahí and BRS Morena cultivars may be due to variations in the water content of the sesame cultivars, affecting saliva production and nutrient intake [25,32]. The shorter and longer duration of the first and fifth instars, respectively, of *N. viridula*, in the three cultivars may be due to the greater nutritional needs of fifth-instar individuals [27]. This is common in Heteroptera with more intense morphophysiological changes as they approach maturity [33] and, in the latter, increasing the need for nutrients to molting and transformation to adults with adequate reproductive potential [34].

The greater weight of *N. viridula* females than males in all treatments is due to a greater accumulation of lipids by the former for reproduction [30,32]. The greater weight of adults of both sexes of *N. viridula* fed with immature green capsules of BRS Morena can be attributed to the higher water content and nutritional quality of this cultivar [12,34]. These variations were also reported for heavier nymphs of this stinkbug fed with soybean pods of the cultivars BR-16, PI 229358 or PI 274454, than those fed with PI 227687 and IAC-100 [34].

The longer pre-oviposition and oviposition periods of *N. viridula* fed with mature sesame capsules, respectively, of the BRS Morena and BRS Anahí may be related to the lower and higher oil content of their seeds. Fat reserves directly affect the energy metabolism of seed-sucking insects, constantly spending energy and needing to feed on seeds with high oil content to accumulate reserves and to survive during periods of food scarcity [35,36].

The greater number of egg masses and eggs per female of *N. viridula* fed with mature capsules of the BRS Anahí confirms its better nutritional quality compared to the others sesame cultivars. These results are similar to and lower than, respectively, the number of egg masses (n = 4.1) and eggs per female (n = 297.9) of this insect fed immature sesame capsules of an unknown variety [11], which was attributed to the morphophysiological differences between those evaluated [13]. This may also explain the higher and lower numbers of eggs per egg masses and eggs, respectively, of each *N. viridula* female fed with mature sesame capsules of the cultivars BRS Anahí and BRS Morena. Changes in the diet of heavier *N. viridula* females fed with BRS Morena and BRS Seda shortly after their emergence as adults reduced their reproductive performance due to a lower nutritional quality of the mature sesame capsules of some cultivars, reducing their lipid storage [25]. On the other hand, the greater number of eggs per mass of this bug fed with BRS Morena and BRS Seda may indicate that malnourished females could compensate their poorer physiological condition by depositing a greater number of eggs per mass, maximizing their reproductive success and ensuring a greater number of offspring [37].

The higher rate of nymph hatching per *N. viridula* female fed with mature capsules of the BRS Anahí sesame cultivar confirms its suitability for the reproduction (higher oil content) of this stinkbug [36]. The lack of nymph hatching with BRS Morena may indicate that this cultivar is a source of resistance to *N. viridula* may be by antibiosis. However, the possibility of non-preference or antixenosis mechanism cannot be excluded, as their effects are difficult to separate in controlled laboratory conditions [34].

The similar longevity between *N. viridula* males and females fed mature capsules of the three sesame cultivars and longer for both sexes with those of BRS Anahí and BRS Morena may be related to the physiological compensation of this bug, with heavier and lighter individuals, respectively, when fed with dry seeds with low and high lipid contents [36,38,39].

## 4. Materials and Methods

### 4.1. Study Location

This study was carried out in the entomology laboratory (7°13′32″ S latitude and 35°54′19″ W longitude) and in the experimental field (7°13′35″ S latitude and 35°54′21″ W longitude) of the Embrapa Algodão in the municipality of Campina Grande, Paraíba state, Brazil from November 2022 to May 2023.

### 4.2. Insects and Plant Material

Specimens of *N. viridula* were collected from a castor bean plantation in the Embrapa Algodão experimental field in September 2022 and reared for five generations in PVC cages, covered at the base with Styrofoam and with voile fabric at the apex. The insects were fed *ad libitum* with green macassar bean pods, *Vigna unguiculata* (L.) Walp. and lima beans, *Phaseolus vulgaris* (L.) (methodology adapted from the work of Corrêa-Ferreira [40]) and kept in a climate-controlled chamber at 25 ± 1 °C, 68 ± 10% relative humidity and a 14 h photophase. Sesame seeds of the BRS Anahí, BRS Morena and BRS Seda cultivars, from the Active Germplasm Bank of Embrapa Algodão, are widely used by small and large rural producers in Mato Grosso state, the largest sesame producer in Brazil [41].

### 4.3. Nymph Bioassays

Sesame seeds of each cultivar were planted in an area of 50 m^2^ (10 × 5 m) at the Embrapa Algodão in September 2022 spaced with 0.70 m and 0.10 m between plants and rows, respectively. Fertilizers were used according to soil analyses and following the technical recommendations for this plant with urea (45% N), phosphorus pentoxide (18% P_2_O_5_) and potassium chloride (60% K_2_O) as NPK sources (Fertilizantes Heringer SA, Paulínia, São Paulo, Brazil), respectively.

In total, 150 *N. viridula* eggs were obtained from the laboratory stock rearing and separated into groups of 50 per treatment, with 10 of them per Petri dish (10 cm × 0.9 cm in diameter and height lined, respectively, with filter paper) until their hatching. The nymphs, immediately hatched, were transferred to plastic pots (10 cm high × 5 cm in diameter) with a lid full of fine perforations to prevent insects from escaping and allow for aeration.

The experimental design used randomized blocks in three treatments with newly emerged nymphs of *N. viridula* fed with green capsules of the sesame BRS Anahí (T1); BRS Morena (T2) and BRS Seda cultivars (T3) and four replications, each with ten newly emerged nymphs per plastic pot. Two to three green sesame capsules were supplied, every two days, per group of ten nymphs until the beginning of the adult stage of this insect per plastic pot. The blocks were considered the different shelves of the acclimatized chamber to reduce the effect of any possible internal temperature gradient from top to bottom. The plastic pots, with the nymphs, were kept in a climate-controlled chamber (25 ± 2 °C, 70 ± 10% relative humidity and 12 h photoperiod) until the adults emerged.

The duration and survival during the first, second, third, fourth and fifth instars and in the nymph stage of *N. viridula* were determined. 

### 4.4. Adult Bioassays

The same experimental design as the nymph bioassay was used for the adults, but the methodology was adapted for this insect stage.

Newly emerged *N. viridula* adults from the nymph bioassay were weighed on an AY220 analytical balance (Shimadzu Corporation, Columbia, MD, USA) with an accuracy of 0.0001 g. Fifteen pairs of *N. viridula* were selected per treatment, individualized in plastic pots for mating and egg laying until the death of the females. These *N. viridula* pairs were fed with 10 dry capsules of the same sesame cultivar and maintained under the same climatic conditions in which their immature stages developed. The number of eggs laid was quantified, daily, at 2:00 P.M. using an EL224 stereomicroscope (BEL Engineering, Monza, Milano, Italy) with 20 × magnification when the reproductive characteristics, sex ratio, incubation period, longevity, fertility, number of egg masses, eggs per laying and viable eggs were determined. 

### 4.5. Sesame Characterization

The water and oil content of immature and mature sesame seed capsules, respectively, were determined to understand how these substances affect food intake by seed-sucking insects. The water content of 50 green sesame capsules and the oil content of 1500 dry seeds, per cultivar, were determined, respectively, using the oven method at 105 °C for 24 h and solvent extraction [42] (Figure 1).

### 4.6. Data Analysis

The normality of the data of the development period, survival and reproductive characteristics of *N. viridula* was verified using the Shapiro–Wilk test and the homoscedasticity of the residuals with the Bartlett test. The data were then submitted to ANOVA and the means compared using the Tukey test (*p* = 0.05). The data were analyzed using the System for Statistical and Genetic Analysis (Viçosa, MG, Brazil) [43].

## 5. Conclusions

This study provides valuable insight to the development, survival and reproduction of *N. viridula* fed on sesame capsules. The survival of nymphs and in each of the five instars of *N. viridula* did not differ with green sesame capsules of the three cultivars evaluated. The duration of the nymph stage of this bug was shorter with green sesame capsules of BRS Morena than with those of BRS Anahí. The oviposition period, the number of egg masses and eggs per female and the percentage of nymph hatching were higher with mature sesame capsules of the BRS Anahí cultivar and lower with those of the other ones. Nymphs did not hatch from eggs deposited by *N. viridula* females fed mature seed capsules of the sesame cultivar BRS Morena. BRS Morena may be a possible resistance source to *N. viridula*.

## Figures and Tables

**Table 1:** Summary model of two-way analysis of variance (ANOVA) for the effects of treatment (sesame cultivars) and instar in the survival and duration of the development of *Nezara viridula* (Hemiptera: Pentatomidae) fed with immature capsules (fruits) of this plant. Campina Grande, Paraíba state, Brazil, 2023.

Source of Variation	Model	DF	Mean Square	*F*	*p*
Survival (%)	Sesame cultivar (SC)	2	301.46	1.90	*p* > 0.05
	Instar (I)	4	887.31	5.58	*p* < 0.01
	SC × I	8	211.66	1.33	*p* > 0.05
	Residue	60	159.06	-	-
Development duration (days)	Sesame cultivar (SC)	2	12.33	6.50	*p* < 0.01
	Instar (I)	4	70.55	37.17	*p* < 0.01
	SC × I	8	2.72	1.43	*p* > 0.05
	Residue	60	1.90	-	-

**Table 2:** Survival and duration (mean ± standard error) of each instar and of the nymph stage of *Nezara viridula* (Hemiptera: Pentatomidae) fed with immature capsules (fruits) of three sesame cultivars at 25 ± 1 °C, 70 ± 10% relative humidity and 14 h photophase. Campina Grande, Paraíba state, Brazil, 2023.

Stage	Instar ^2^	Treatments—Sesame Cultivars ^1^		
		BRS Anahí	BRS Morena	BRS Seda
Survival (%)	First	100.00 ± 0.00 A a	100.00 ± 0.00 A a	100.00 ± 0.00 A a
	Second	94.00 ± 3.22 A ab	96.00 ± 2.86 A ab	100.00 ± 0.00 A ab
	Third	73.83 ± 5.78 A c	77.00 ± 9.30 A c	92.00 ± 1.43 A c
	Fourth	90.44 ± 3.42 A abc	93.50 ± 3.49 A abc	86.89 ± 3.26 A abc
	Fifth	89.72 ± 3.68 A bc	74.29 ± 9.20 A bc	94.92 ± 2.73 A bc
	Nymph	56.00 ± 5.01 A	52.00 ± 8.23 A	76.00 ± 3.94 A
Duration (days)	First	3.02 ± 0.00 Ac	2.40 ± 0.21 Bc	3.00 ± 0.01 ABc
	Second	7.98 ± 0.43 Ab	6.15 ± 0.66 Bb	6.56 ± 0.43 ABb
	Third	8.36 ± 0.47 Ab	4.81 ± 0.22 Bb	5.96 ± 0.73 ABb
	Fourth	6.48 ± 0.35 Ab	5.63 ± 0.07 Bb	6.44 ± 0.39 ABb
	Fifth	9.18 ± 0.34 Aa	8.47 ± 0.63 Ba	8.37 ± 0.67 ABa
	Nymph	39.30 ± 0.76 A	34.46 ± 0.46 B	36.38 ± 1.06 AB

Means followed by the same uppercase letter, in the line per treatment ^1^, and lowercase letter, in the column per instar ^2^, do not differ by the Tukey test at 5% probability.

**Table 3:** Reproductive characteristics (mean ± standard error) of *Nezara viridula* (Hemiptera: Pentatomidae) adults fed with mature seeds of three sesame cultivars at 25 ± 1 °C, 70 ± 10% relative humidity and photophase of 14 h. Campina Grande, Paraíba state, Brazil, 2023.

Variables	Treatments—Sesame Cultivars ^1^		
	BRS Anahí	BRS Morena	BRS Seda
Preoviposition (days)	13.20 ± 1.23 b	52.00 ± 0.00 a	30.33 ± 8.34 ab
Oviposition (days)	22.80 ± 2.31 a	1.00 ± 0.00 b	2.67 ± 1.28 b
Postoviposition (days)	21.89 ± 5.34 a	6.56 ± 0.00 a	12.47 ± 1.28 a
Egg masses per female	4.50 ± 0.35 a	1.00 ± 0.00 b	1.33 ± 0.16 b
Eggs per laying	26.06 ± 1.52 b	42.00 ± 0.00 a	66.33 ± 0.79 a
Eggs per female	119.57 ± 11.26 a	77.00 ± 0.00 b	87.33 ± 14.88 b
Egg hatching (%)	77.37 ± 3.48 a	0.00 ± 0.00 b	34.10 ± 8.80 b
Longevity (days) (♀) ^2^	57.89 ± 5.42 aA	59.56 ± 5.72 aA	45.47 ± 2.87 bA
(♂)	54.33 ± 4.62 aA	51.67 ± 3.46 aA	34.13 ± 3.81 bA

Means followed by the same lowercase letter, in the line per treatment ^1^, and capital letter, in the column for longevity per sex ^2^, do not differ by the Tukey test at 5% probability. Means transformed into × root + 0.5 for statistical analysis purposes ^1^. Original means are shown.

## Data Availability

Data will be made available on request.
